# Draft genome sequence of *Aeromonas enteropelogenes* A5 isolated from diseased tiger shrimp (*Penaeus monodon*) in Bangladesh

**DOI:** 10.1128/mra.00005-26

**Published:** 2026-03-30

**Authors:** Md Rony Babu, Ms Khadiza Sarkar, Sulav Indra Paul, Bishwajit Karmakar Sunny, Umma Habiba Tanjum, Farzana Yeasmin, Arpita Guha, Promi Saha, Santu Biswas, Md. Abdul Hannan, Tofazzal Islam, Md Mahbubr Rahman

**Affiliations:** 1 Institute of Biotechnology and Genetic Engineering, Gazipur Agricultural University 198780, Gazipur, Bangladesh; 2 Institute for Biosecurity and Microbial Forensics, Oklahoma State University 7618, Stillwater, Oklahoma, USA; 3 Department of Biological, Geological, and Environmental Science, Cleveland State University 2564, Cleveland, Ohio, USA; 4 Department of Aquatic Animal Health Management, Faculty of Fisheries and Marine Science, Sher-e-Bangla Agricultural University, Sher-e-Bangla Nagar 54494, Dhaka, Bangladesh; University of Maryland School of Medicine, Baltimore, Maryland, USA

**Keywords:** *Aeromonas enteropelogenes*, *Penaeus monodon*, shrimp disease, virulence-associated gene

## Abstract

We report the draft genome sequence of *Aeromonas enteropelogenes* A5, isolated from a diseased shrimp (*Penaeus monodon*) in Bangladesh. The 4,571,270 bp genome contains 4,125 predicted coding sequences with 59.6% GC content and multiple putative virulence-associated genes. The genome provides insights into the pathogenic potential of *A. enteropelogenes* in shrimp aquaculture.

## ANNOUNCEMENT

*Aeromonas* spp. are Gram-negative, facultative anaerobic bacteria ubiquitous in aquatic environments (1). The genus comprises more than 30 species, many of which are recognized as opportunistic pathogens of fish, shrimp, animals, and humans (2, 3). Notably, *Aeromonas enteropelogenes* is considered synonymous with *Aeromonas torta* (4). Here we report the draft genome sequence of *A. enteropelogenes* strain A5, isolated from diseased shrimp.

Diseased shrimps were collected from a gher (shrimp farm) in Shamnagar, Satkhira (23.9999° N, 90.4203° E), Bangladesh. The infected shrimp exhibited lethargy, reddish body, and pale hepatopancreas. The shrimp surfaces were disinfected with 70% ethanol, and the hepatopancreas was aseptically removed, homogenized in sterile physiological saline, and subjected to 10-fold serial dilutions. Thirty microliter aliquots of each dilution were spread onto TCBS agar (Liofilchem S.r.l., Italy) and incubated at 25°C for 24 h. A single colony from the 10^−3^ dilution, designated as strain A5, was purified through successive subculturing on nutrient agar (Liofilchem S.r.l.). For genomic DNA extraction, a single colony was inoculated into 5 mL of nutrient broth (Liofilchem S.r.l.) and incubated overnight at 30°C with shaking at 200 rpm. Genomic DNA was extracted using the GeneJET genomic DNA purification kit (Thermo Fisher Scientific, USA) (5). DNA quality and concentration were assessed using a NanoDrop spectrophotometer (Thermo Fisher Scientific). Sequencing libraries were prepared using the Illumina Nextera XT kit (6). One nanogram of genomic DNA was fragmented using 5 μL of Tagment DNA enzyme and 10 μL of buffer (Illumina, Inc., San Diego, CA, USA) at 55°C for 10 min, followed by neutralization with 5 μL Neutralize Tagment buffer (7). A 12-cycle PCR was done for barcoding nucleotide sequences (7). Indexed DNA fragments were purified with AMPure XP beads (Beckman Coulter, Inc., Australia), normalized to 5 nM, denatured with 0.2 N NaOH, diluted to 13 pM, and sequenced on an Illumina MiSeq platform (600-cycle run).

Raw reads (410,692) with a coverage of 30.0× were quality-filtered using Trimmomatic v0.38 and PRINSEQ v0.20.3 (8, 9). *De novo* assembly was performed using SPAdes v3.13.1 (10) and validated with QUAST v5.0.2 (11). The NCBI Prokaryotic Genome Annotation Pipeline v6.10 (12) was used for genome annotation, which identified 4,279 total genes, including 4,125 protein-coding genes and 118 RNA genes (104 tRNAs, 10 rRNAs, and 4 ncRNAs), along with 36 pseudogenes (Table 1).

**TABLE 1:** Genome assembly and annotation statistics of *Aeromonas enteropelogenes* strain A5

Features	Value
Genome size (bp)	4,571,270
Number of contigs	83
GC content (%)	59.6
N50 (bp)	245,669
L50	6
Total genes	4,279
Protein-coding sequences	4,125
Protein-coding genes	4,125
Total RNA genes	118
tRNA genes	104
rRNA genes	10
ncRNA genes	4
Pseudogenes	36
Annotation pipeline	NCBI Prokaryotic Genome Annotation Pipeline v6.10

Screening for virulence genes, potential antibiotic resistance genes, and plasmids was performed using VirulenceFinder v2.0 (13), ResFinder v4.0 (14), and PlasmidFinder v2.0 (15), respectively. Pathogenicity prediction for human hosts was evaluated using PathogenFinder v1.1 (16). Default parameters were used for all software unless otherwise specified. A macrolide resistance gene (*blaTRU*) was identified with 97.04% identity. Multiple virulence-associated genes were detected (e.g., *flmH*, *tapF*, *vasH*, *tapV*, *flrA*, *cyaB*, *bvgS*, *pvdE*, *fleQ*, and *rtxB*), while no plasmid replicons were found. PathogenFinder estimated a 0.433 probability of human disease association, suggesting a limited direct public health risk.

The genome sequence provides a genomic foundation for understanding the virulence mechanisms of *A. enteropelogenes* A5 and aids in developing diagnostic and disease management strategies for shrimp aquaculture.

## Data Availability

The raw data are accessible under accession number JBFQEF000000000, and the version described in this paper is version JBFQEF010000000. The data can be found under BioSample accession number SAMN42483725 and BioProject accession number PRJNA1135474. The raw data are available from the Sequence Read Archive under accession number SRX31866214.
